# Augmentation of Fear Extinction by Transcranial Direct Current Stimulation (tDCS)

**DOI:** 10.3389/fnbeh.2018.00076

**Published:** 2018-04-25

**Authors:** Natalie Dittert, Sandrina Hüttner, Thomas Polak, Martin J. Herrmann

**Affiliations:** Department of Psychiatry, Psychosomatics, and Psychotherapy, Center of Mental Health, University Hospital Würzburg, Würzburg, Germany

**Keywords:** fear conditioning, brain stimulation, tDCS, skin conduction response, ventromedial prefrontal cortex

## Abstract

Although posttraumatic stress disorder (PTSD; DSM-V 309.82) and anxiety disorders (DSM-V 300.xx) are widely spread mental disorders, the effectiveness of their therapy is still unsatisfying. Non-invasive brain-stimulation techniques like transcranial direct current stimulation (tDCS) might be an option to improve extinction learning, which is a main functional factor of exposure-based therapy for anxiety disorders. To examine this hypothesis, we used a fear conditioning paradigm with female faces as conditioned stimuli (CS) and a 95-dB female scream as unconditioned stimulus (UCS). We aimed to perform a tDCS of the ventromedial prefrontal cortex (vmPFC), which is mainly involved in the control of extinction-processes. Therefore, we applied two 4 × 4 cm electrodes approximately at the EEG-positions F7 and F8 and used a direct current of 1.5 mA. The 20-min stimulation was started during a 10-min break between acquisition and extinction and went on overall extinction-trials. The healthy participants were randomly assigned in two double-blinded process into two sham stimulation and two verum stimulation groups with opposite current flow directions. To measure the fear reactions, we used skin conductance responses (SCR) and subjective ratings. We performed a generalized estimating equations model for the SCR to assess the impact of tDCS and current flow direction on extinction processes for all subjects that showed a successful conditioning (*N* = 84). The results indicate that tDCS accelerates early extinction processes with a significantly faster loss of CS+/CS– discrimination. The discrimination loss was driven by a significant decrease in reaction toward the CS+ as well as an increase in reaction toward the CS– in the tDCS verum groups, whereas the sham groups showed no significant reaction changes during this period. Therefore, we assume that tDCS of the vmPFC can be used to enhance early extinction processes successfully. But before it should be tested in a clinical context further investigation is needed to assess the reason for the reaction increase on CS–. If this negative side effect can be avoided, tDCS may be a tool to improve exposure-based anxiety therapies.

## Introduction

Posttraumatic stress disorder (PTSD; DSM-V 309.81) and anxiety disorders (DSM-V 300.xx) have a high 12-month-prevalence of 16% (PTSD 2%, anxiety disorder 14%) in Europe and belong thereby to the most common psychiatric diseases (Wittchen et al., 2011). Cognitive behavioral therapy with exposure elements is, amongst others, the recommended treatment form for PTSD and many entities of anxiety disorders, namely specific phobia (DSM-V 300.29), social anxiety disorder (DSM-V 300.23), generalized anxiety disorder (DSM-V 300.xx), panic disorder (DSM-V 300.01), and agoraphobia (DSM-V 300.22; Rauch et al., 2012; Bandelow et al., 2014). The development of this therapy is based on an explanatory cognitive and learning model of anxiety. Such models assume that processes of classical conditioning result in the development of anxiety. Classical fear conditioning is an associative learning process, which links harmless stimuli with fearful experiences. Anxiety toward these former harmless stimuli is then preserved by processes of operant conditioning, which lead to avoidance behavior. This avoidance behavior prevents the ability for other associations to be made, leaving the fear intact (Mowrer, 1956). Therefore, new neutral experiences with the former harmless stimuli lead to a reduction of the fearful association (Myers and Davis, 2002). According to classical conditioning, this process is called extinction learning. As extinction learning can lower anxiety through the diminishing of fearful associations it was used as a basis for the development of exposure therapies for anxiety disorders (McNally, 2007). Hence, finding a method that improves extinction processes is a good starting point to discover a targeted modulation procedure for enhancing exposure therapies. One option could be the use of non-invasive brain-stimulation techniques (Bajbouj and Padberg, 2014; Marin et al., 2014).

Non-invasive brain-stimulation works through changing the activity of several brain areas by application of magnetic fields or currents with devices that are placed on the exterior of the head. Thereby non-invasive brain-stimulation can support the effect of psychotherapy (Bajbouj and Padberg, 2014) as psychotherapy also changes several neuronal structures and their activity like Beauregard (2014) has proven for anxiety disorders. One non-invasive stimulation technique, that is already recommended for the treatment of unipolar depression in Germany, is repetitive transcranial magnetic stimulation (rTMS; DGPPN, November 2015)^1^. Besides depression, recent studies showed that rTMS could successfully improve extinction processes and lower anxiety as well. Guhn et al. (2014) stimulated the ventromedial prefrontal cortex (vmPFC) with rTMS prior to extinction learning and could achieve a successful improvement of early extinction learning and extinction recall. Further investigation revealed the effects of Guhn's stimulation protocol for the improvement of exposure therapy of acrophobic patients (Herrmann et al., 2017). Raij et al. (2017) tried a temporally specific rTMS of the prefrontal cortex according to a prior study, which showed, that stimulation of the infralimbic cortex of rats—the equivalent to the human vmPFC—only improves extinction when it is applied 100 ms after the stimulus onset (Milad et al., 2004). Raij et al. (2017) used an incomplete extinction paradigm with just four trials and could achieve an improvement of extinction recall on the next day. They made no measurements during extinction learning, thus, rTMS could have affected extinction recall or the four trials of early extinction learning equally.

Transcranial direct current stimulation (tDCS), which we used in this study, is another important non-invasive brain stimulation technique that receives increasing attention in psychiatric research. In comparison to rTMS, tDCS is more pleasant, has less aversive side effects and its application is easier and cheaper (Poreisz et al., 2007). tDCS is applied using two electrodes placed around the stimulation area on the scalp. The current flows from anode to cathode and passes through all brain areas that are located between the electrodes, thus, the focal specification is not very high. Overall, a subthreshold activation of brain areas near the anode and a deactivation near the cathode is generated (Nitsche and Paulus, 2000). To be more precise the orientation of the cell axes in relation to the current flow is important for the outcoming activation. Cell parts nearer the anode become hyperpolarized, those nearer the cathode depolarized (Bikson et al., 2004). A successful increase in brain activation can usually be obtained with a somatic depolarization and a terminal hyperpolarization. So, pyramidal cells in cortical regions that lie parallel to the scalp near the anode get thereby activated by these so-called radial current proportions (Rahman et al., 2013). On the contrary, tangential current proportions, that flow parallel and not vertical to the cortical surface, do not activate entire cortical areas, but rather several corticocortical afferent nerve pathways or single axon terminals (Rahman et al., 2013). Especially the effect of tangential current flow proportions cannot be predicted precisely. The angle in which the current meets the cortex and individual anatomical factors such as cortical folding must be considered as well (Bikson et al., 2013). Additionally, there are activity- and input-selective mechanisms (Bikson et al., 2013). So, the task, which is done during stimulation, influences the brain activity, too (Reato et al., 2010). Further, tDCS improves the processing of some contents but this goes usually at the expense of other contents, whose reception decreases parallelly (Bikson et al., 2004). In addition to the direct activation of several brain areas, tDCS seems to modulate the dopamine secretion (Tanaka et al., 2013; Broeder et al., 2015; Agarwal et al., 2016).

Even though the focal specification of tDCS is rather low, a specific electrode placement can get several brain areas into the focus of stimulation. So, the underlying neuronal processes that are associated with a specific psychiatric disease determine a therapeutic reasonable stimulation aim in the brain and lead thereby the decision for the electrode positions. Hence, for the alleviation of anxiety disorders, adequate stimulation aims can be derived from the underlying neuronal mechanism of exposure therapies, which is extinction learning. One important brain area for extinction processes is the vmPFC. Results of functional resonance imaging (fMRI) proved a heightened activation of the vmPFC during extinction learning (Gottfried and Dolan, 2004) and a decrease in vmPFC depression during progressive extinction learning as well as a correlation of vmPFC activation and extinction retention (Phelps et al., 2004). Consistent with these findings the vmPFC activity increased during late extinction learning in near-infrared spectroscopy (NIRS; Guhn et al., 2012). Furthermore, lesions or pharmacological deactivation of this brain area led to an impairment of extinction consolidation and recall in rats (Quirk et al., 2000; Morgan et al., 2003; Sierra-Mercado et al., 2006). Apart from the vmPFC, the amygdala plays a vital role according to extinction processes (Knight et al., 2004; Sotres-Bayon et al., 2007; Herry et al., 2008). Herry et al. (2008) found special extinction neurons in the amygdala that were activated during extinction learning. To close the circle, these neurons showed also strong bidirectional connections with the vmPFC. Based on these data we aimed—like many other researchers, who tried to modulate extinction processes via brain stimulation—to activate the vmPFC (Guhn et al., 2014; Abend et al., 2016; Van't Wout et al., 2016, 2017; Raij et al., 2017). On the neurobiological level, especially the neurotransmission of dopamine seems to be a crucial factor for functioning extinction (Hikind and Maroun, 2008; Raczka et al., 2011; Abraham et al., 2016).

About the effects of tDCS on extinction mainly two researchers, R. Abend and M. van't Wout, have published so far, though none of their studies could substantially improve extinction processes. Abend et al. (2016), who placed the anode on the forehead and the cathode on the back of the head and stimulated parallel to extinction learning, did a 3-day fear conditioning paradigm with extinction learning and recall on different days. They found no improvement of extinction but anxiety generalization effects with increased reactions on CS– in their tDCS condition and a fear potentiation toward the CS+ in their alternating current condition. As probable causes for these effects the authors considered on the one hand the unintentional stimulation of dorsomedial brain areas and on the other hand that the stimulation was not temporally specific, which seemed to be important in Milad et al. (2004) as mentioned above. Van't Wout published twice exploring a cross-over-design with the anode on the left forehead on EEG-Position AF3 and the cathode on the contralateral mastoid. She used a 2-day paradigm with conditioning and extinction learning on the first and extinction recall on the second day. In 2016 she found a slight improvement of late extinction learning in healthy participants when the stimulation took place during early extinction learning, but this effect could not be distinguished from anxiolytic tDCS-aftereffects with certainty (Van't Wout et al., 2016). In 2017 she stimulated PTSD patients during or after extinction learning and found only a trend-significant improvement of extinction recall in the after extinction learning condition (Van't Wout et al., 2017). Both authors aimed to stimulate the vmPFC but used different electrode positions to do so. As mentioned above the angle in which the current meets the cortical surface affects the stimulation effects, too. On the one hand this could be a reason for their distinct results, but on the other hand, it leaves hope that the investigation of further electrode positions could finally lead to a successful improvement of extinction processes. Therefore, we want to go on finding a tDCS-protocol that can substantially improve extinction processes by increasing the activation of the vmPFC.

Furthermore, we wanted to assess the effect of the current flow direction. Opposite current flow directions in a bitemporal electrode placement do not determinedly lead to opposite tDCS effects. The outcome can rather be distinct because of the complex tDCS mechanisms, which include e.g., parameters of cortical folding and changes in the dopamine secretion as already said. Additionally, lateral cortical areas get affected by right or left anodal tDCS in diverse ways, whereas our actual stimulation aim, the vmPFC, was equidistantly located between anode and cathode. Thus, we expected that both current flow directions would lead to similar activation patterns in the vmPFC region but have different effects on other prefrontal cortical areas, which may affect our outcome measures. According to the functional diversity of the two hemispheres, both current flow direction can have advantages and disadvantages. The right lateral prefrontal cortex seems to be important for emotional regulation processes (Klumpers et al., 2010; Herrmann et al., 2016a). Therefore, right anodal stimulation, which rather activates the right hemisphere, could lead to stronger extinction learning. But on the other hand, patients with anxiety disorders show a decreased left cortical activation (Thibodeau et al., 2006), thus, it is conceivable that the increase of left cortical activity by left anodal tDCS might reduce anxiety and improve extinction learning as well. Investigating the effects of the current flow direction in this context seemed to be very interesting but difficult to predict at the same time. Therefore, we could not make any certain predictions about its effect in advance, but we expected that right and left anodal stimulation would not result in similar effects.

As the main effect of our tDCS-protocol we expected—according to the successful improvement of early extinction learning by rTMS by Guhn et al. (2014)—an improvement in extinction learning most notably in early extinction processes. To avoid anxiety generalization effects, as in Abend et al. (2016), we chose a bitemporal electrode positioning which ensured a recess of fear-generating dorsomedial brain areas.

## Materials and methods

### Participants

One hundred and thirty one healthy participants were recruited through online displays and randomly and double-blinded assigned into four groups: two real-stimulation groups with right vs. left anodal stimulation and two sham-stimulation groups. Blinding worked through a random assignment of codes to each participant via code lists, which were separated by sex and current flow direction. The investigator keyed in these codes into the stimulation device, which then decided if the participants received sham- or real-stimulation. Therefore, it was necessary to collect data from two separate sham groups (right or left anodal) to uphold the blinding of the investigator until the end of data collection.

All subjects gave self-disclosure about the main inclusion criteria, which were no psychiatric or neurological diseases (especially no epilepsy or elevated brain pressure) now or earlier, no current heart disease or hearing loss, age between 18 and 35 years, right-handedness, no metal or cochlear head-implants and no recent consumption of psychotropic drugs. To control hormonal levels, an additional inclusion criterion for women was being in the intake-phase of hormonal contraceptives (cf. Guhn et al., 2012), whereas pregnancy or current breastfeeding were exclusion criteria. In total 47 participants were excluded, most of them (36) because of insufficient fear conditioning. Sufficient fear conditioning was defined by a higher skin conductance response (SCR) toward the conditioned stimulus (CS+) that was paired with the unconditioned stimulus (UCS), compared to the unpaired conditioned stimulus (CS–) during the last two acquisition trials. Other reasons for exclusion were high depression scores (Allgemeine Depressionsskala in Kurzform; ADS-K; Hautzinger and Bailer, 1993 ADS-K > 16; *N* = 8), technical problems (*N* = 1), early termination at own request (*N* = 1), and undetectable SCR response after a deep breath at the beginning of measurements (*N* = 1). Finally, 84 participants remained for the analysis. For these remaining subjects, no significant group differences for age, gender, body size (weight, height, and head size), drug use (caffeine, nicotine, cannabis), trait-anxiety (state and trait anxiety inventory, form X2; STAI-X2; Laux et al., 1981), anxiety sensitivity (anxiety sensitivity index 3; ASI-3; Taylor et al., 2007), depression (ADS-K), and negative and positive affect (positive and negative affect scale; PANAS; Watson et al., 1988) could be found in statistical group comparisons with generalized estimating equation models (GEEs) (see Table 1). After participants were given a complete description of the study and its procedures, written informed consent was obtained in accordance with the Declaration of Helsinki in its latest version. All procedures were approved by the ethics committee of the medical faculty of the University of Würzburg. All subjects participated voluntary and received an expense allowance of 15 euros.

**Table 1:** Sample description.

	**Sham**				**Real**			
	**Right anodal (*****N*** = **17, 10** **♀** **)**		**Left anodal (*****N*** = **17, 9** **♀** **)**		**Right anodal (*****N*** = **26, 14** **♀)**		**Left anodal (*****N*** = **24, 13** **♀)**	
	***M***	***SD***	***M***	***SD***	***M***	***SD***	***M***	***SD***
Age (years)	24.0	3.9	25.3	4.1	24.4	4.4	23.3	3.6
Height (cm)	171.7	10.2	174.5	9.0	175.7	7.7	171.7	8.8
Weight (kg)	66.2	13.7	67.6	11.2	67.5	10.5	67.3	10.6
BMI (kg/cm2)	22.2	2.9	22.1	2.2	21.8	2.2	22.8	3.0
Head size (cm)	55.8	2.0	55.9	2.0	56.0	1.7	55.9	1.7
EIH	0.8	0.2	0.8	0.2	0.9	0.1	0.8	0.2
ASI-3	18.1	8.8	14.2	11.9	15.6	8.7	18.0	6.7
ADS-K	7.8	4.1	5.2	4.1	6.6	3.5	6.7	4.2
**STAI**								
Trait	33.9	6.3	32.3	6.9	31.7	6.4	32.7	6.7
State t1	41.4	8.1	37.7	7.1	38.5	8.2	39.0	9.9
State t2	33.8	4.5	33.7	6.1	35.0	5.5	31.1	5.7
**PANAS**								
PA baseline	35.2	6.0	35.8	5.7	36.8	5.5	37.3	5.7
PA t1	26.9	4.7	28.2	5.8	28.7	7.3	29.6	5.7
PA t2	27.6	6.3	29.3	5.7	27.5	5.5	30.4	6.3
NA baseline	17.9	5.1	16.4	5.0	16.6	4.2	16.0	3.9
NA t1	15.1	4.4	14.0	5.8	14.5	3.9	14.3	4.0
NA t2	11.1	1.5	11.6	3.5	11.8	3.5	10.7	1.0

*Displayed are means and standard deviations of sample characteristics and questionnaire scores for sham and real stimulation groups separated for the current flow direction. M, mean; SD, standard deviation; EIH, Edinburgh Inventory of Handedness; ASI-3, Anxiety Sensitivity Index 3; ADS-K, depression score; STAI, State Trait Anxiety Inventory with trait-scale and state-scale after acquisition (t1) and after extinction (t2); PANAS, Positive and Negative Affect Schedule with baseline over the last 12 months and actual score after acquisition (t1) and after extinction (t2); PA, positive affect; NA, negative affect*.

### Stimulation

**Figure 1:**
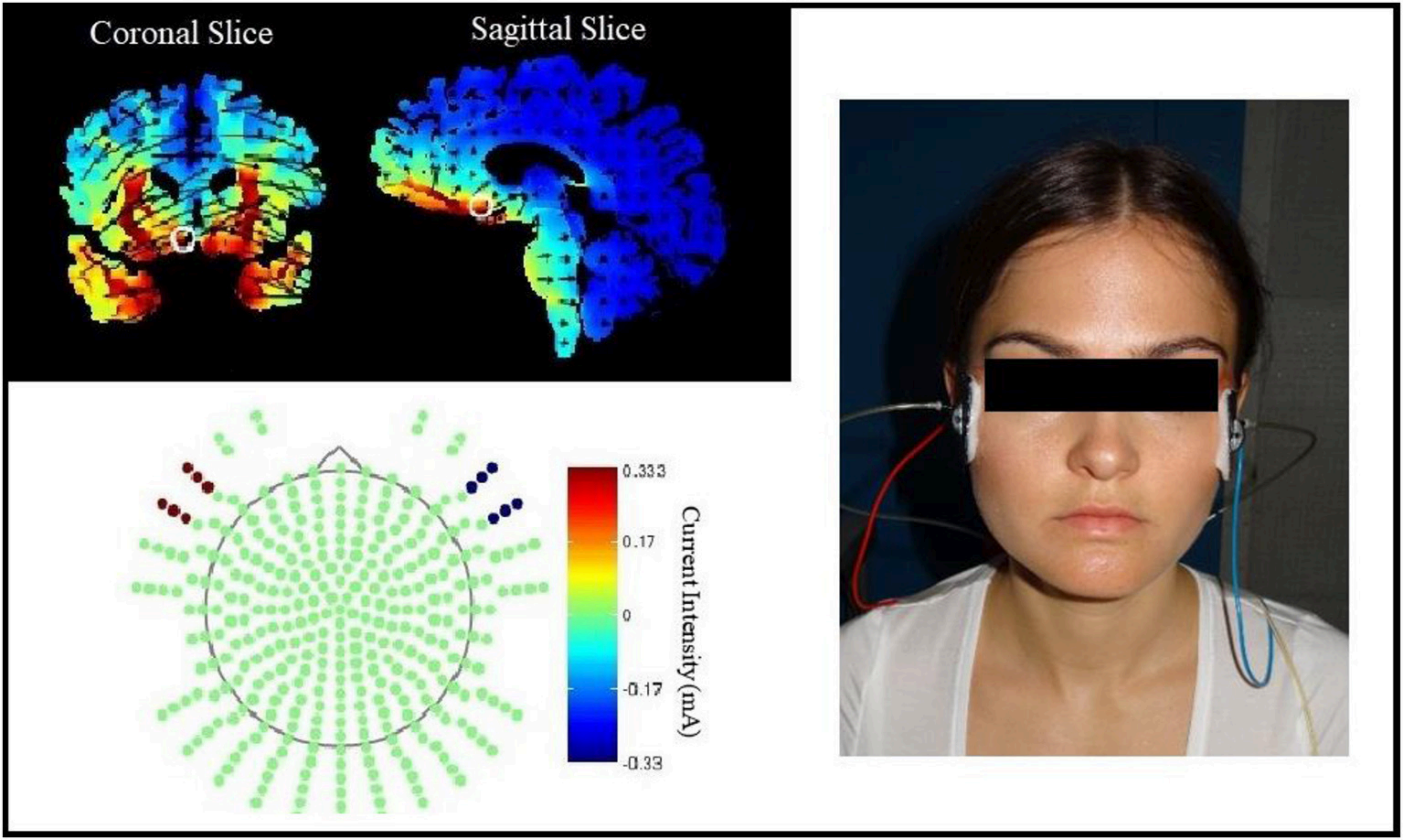
Electrode position selection. The current intensity modeling with HD Explore by Soterix Medical led to the selection of electrode positions near EEG-positions F7 and F8. The figure on the left side shows the modeling for left anodal current flow, the picture on the right shows the actual electrode placement on a participant for right anodal current flow.

tDCS was applied by a battery powered stimulator (Eldith DC-Stimulator, NeuroConn, Ilmenau, Germany) using two approximately 4 × 4 cm rubber electrodes coated with electrode gel (TEN20 conductive neurodiagnostic electrode paste). The stimulation started during a 10-min break between acquisition and extinction and went on until the end of extinction. The real-stimulation protocol had a duration of 1,200 s on a constant level of 1.5 mA and a fade-in and fade-out phase of 10 s each during which the current was slowly turned on in the beginning and off in the end. The sham-stimulation protocol had the same fade-in and fade-out phases, but the constant current phase was shortened to 40 s. The electrode positions were selected by the support of the computer program HD explore by Soterix Medical 3.2 (Kempe et al., 2014), which simulates the brain activation of different stimulation protocols. Our aim was to achieve an intense stimulation of the vmPFC, whereas fear generating dorsomedial brain areas like the dorsal anterior cingulate cortex should be spared out. We chose the positions M20, M21, I20, I21, J13, and J14 for the left and M9, M10, I9, I10, J6, and J7 for the right electrode pad in the 332+4 electrodes model of the HD explore software (see Figure 1). To simplify the electrode application, we calculated the distance of these electrode positions from easily measurable points of the EEG-10-20-system for each participant's head circumference. The final positions were slightly below the EEG-10-20 positions F7 and F8.

The two experimental groups were treated with the same electrode positions but opposite current flow directions. Figure 1 shows the HD explore modeling only for the left anodal stimulation because the activity in the vmPFC, our main stimulation aim, looked similar in modeling for right or left anodal current flow. As mentioned in the introduction, the background of the current flow direction effects is complex and escapes thereby activity modeling.

### Fear conditioning

#### Stimuli

According to Lau et al. (2008), two neutral looking female phases of the NimStim Face Stimulus Set (03F_NE_C and 10F_NE_C; Tottenham et al., 2009) were used as CS. One of these faces was randomly selected as a CS+ and followed by the UCS in the acquisition phase, the other one functioned as a CS– and was never paired with the UCS. As UCS a 95-dB loud female scream simultaneously presented with a fearful expression of the CS+ face was used (sound: FemScream2, no. 276 of the International Affective Digitized Sounds; Bradley and Lang, 1999; pictures: 03F_FE_O or 10_FE_O of the NimStim Face Stimulus Set; Tottenham et al., 2009). The sound was applied via in-ear-headphones (3M E-A-RTONE™ GOLD 3A Insert Earphone with natus® neurology attachment; Natus Europe GmbH).

#### Task

**Figure 2:**
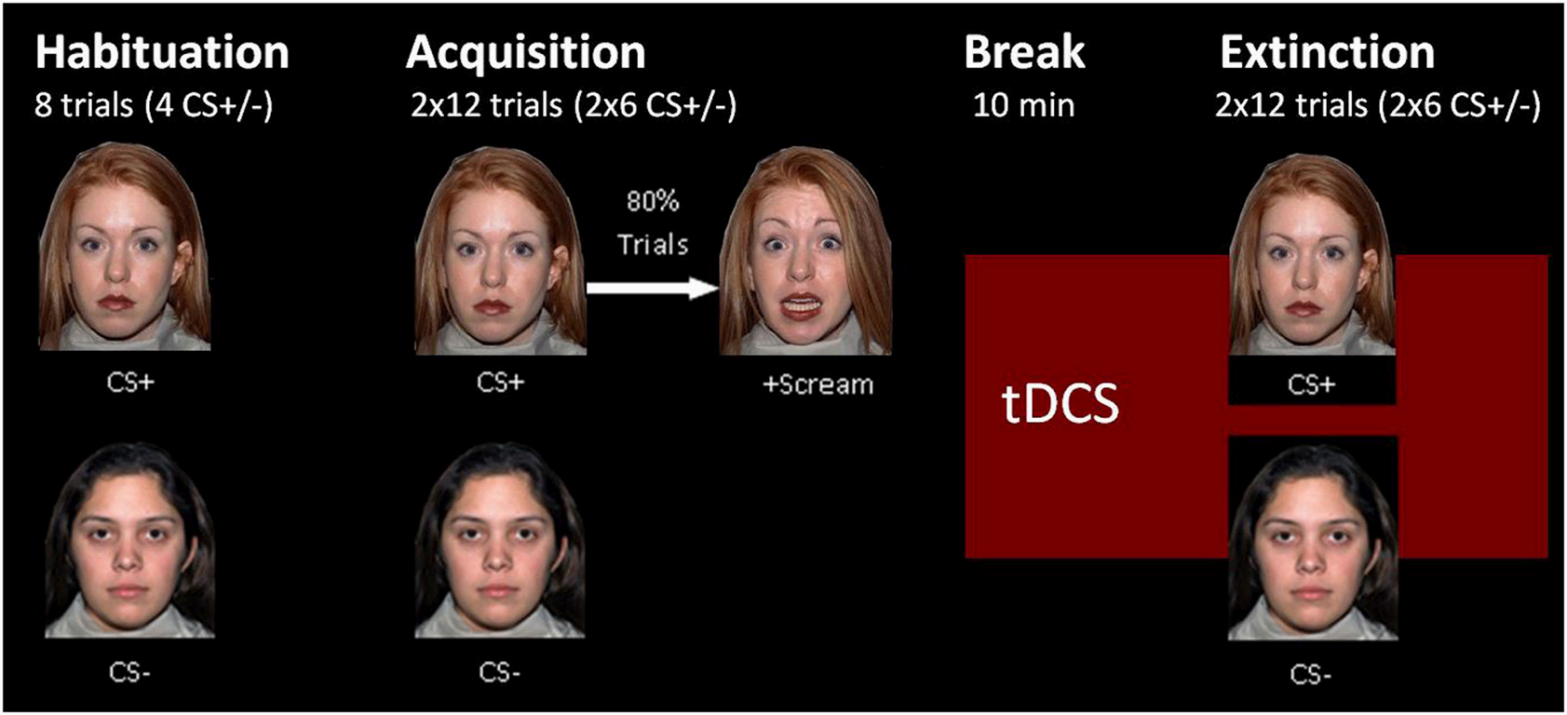
Fear conditioning paradigm.

The experiment was designed with Presentation® software (version 16.5, Neurobehavioral Systems, Inc., Berkeley, CA, www.neurobs.com) and consisted of a habituation phase, two blocks of acquisition and two blocks of extinction, that all took place on the same day (see Figure 2). We separated the extinction phase into two blocks to differentiate between early and late extinction learning and to perform a subjective rating about arousal and valence of both CSs after early extinction learning. The acquisition was split into similar blocks to ensure a regular experimental schedule for the subjects. Both CSs were presented for 6 s in a pseudo-randomized order ensuring a maximum of 2 consecutive presentations of the same stimulus. The intertrial interval had a randomized duration of 9 to 12 s. Whereas, the habituation phase consisted of four presentations of each CS, in every block of the acquisition and extinction phase each CS was shown six times. During the acquisition phase the CS+ was followed by the UCS in five of the six trials per block, so the reinforcement rate was about 80% similar to Abend et al. (2016). We chose a partial reinforcement as it prolongs the process of extinction learning compared to a continuous CS-UCS-pairing (Hilton, 1969; Schurr and Runquist, 1973). Thus, hoping to have more time to detect effects during extinction learning.

### Measurements

#### Questionnaires

To assess the baseline criteria, we measured handedness (Edinburgh inventory of handedness; EIH; Oldfield, 1971), depression (ADS-K), trait anxiety, anxiety sensitivity, and positive and negative affect during the last 12 months and asked for sociodemographic data before the experiment started (for a specification of these questionnaires see section Participants). Additionally, we wanted to assess the change of state anxiety and affect in the course of extinction and questioned them after acquisition and extinction using the STAI-X1 (state and trait anxiety inventory, form X1; Laux et al., 1981) and PANAS forms.

#### Subjective ratings

Arousal, valence, and CS-UCS-contingency were repeatedly rated during the experiment for both CSs. The ratings for arousal and valence were performed on a 9-step visual analog Likert-scale (arousal: from very calm to very exciting, valence: from very pleasant to very unpleasant) after each experimental block. Because extinction learning should not be influenced by artificially induced contingency-attention within extinction processes, the ratings for the CS-UCS-contingency took place after every block except the first extinction block. The contingency was rated on an 11-step visual analog Likert-scale, that ranged from 0 to 100%. Participants were asked how likely they would expect a sound after the CS-pictures.

#### SCR

SCR was recorded with two 5 mm Ag/AgCl surface electrodes placed on thenar and hypothenar of the left hand. The electrodes were connected to an amplifier and recorded via BrainVision Recorder (version 1.20.0701, Brain Products GmbH) in DC mode at a sampling rate of 500 Hz, a range of ± 5,000 mV, and a high cutoff filter of 1,000 Hz. A gradient of 25 mv/μS was used.

SCR analysis was performed with the program PsPM 3.1.1 (http://pspm.sourceforge.net/) using its *general linear model* (GLM) for SCR, which was designed for evoked responses, but is appropriate for event-related SCR with short inter stimuli intervals as well (Bach et al., 2009). The GLM has a high predictive validity for trial-by-trial analysis of SCR data (Bach et al., 2013) and a higher predictive validity than other SCR-analysis methods like the continuous decomposition analysis by Ledalab or conventional peak-scoring (Bach, 2014; Staib et al., 2015). PsPM uses a linear model based approach like fMRI models and calculates beta-estimates for the sympathetic arousal of different experimental conditions, which are defined as regressors. We used one regressor for all UCSs together and built single regressors for each following pair of CS+ or CS– trials, thus, e.g., SCR data for the first and the second CS+, which were presented during extinction learning, built together one regressor. The summation of two following trials to one regressor heightened the predictive validity of our model, which is claimed to be lower for single trials (Bach et al., 2013). For the model calculations, we chose PsPM's *skin conductance response function* (“number 1”), which includes the SCR and its temporal invariants. As PsPM filters data during its processing, we decided to keep the default filter settings (downsampling to 10 Hz, unidirectional first order Butterworth high and low pass filter on a cut-off-frequency of 0.05 and 5 Hz). After model calculation, the statistics of all regressors were exported for statistical analyses. Because SCR data usually show large individual differences, a standardization is useful for the performance of interindividual comparisons (Boucsein et al., 2012). We z-normalized our data as z-standardization seems to be more advantageous in comparison to other standardization methods for SCR data (Ben-Shakhar, 1985).

### Procedure

After completion of written informed consent, the participants filled out questionnaires for baseline measurements and the electrodes for tDCS and SCR were applied. The experiment took place in a darkened and soundproof investigation chamber, in which the subjects were placed alone. As a task description, participants were told that photographs and sounds would be presented and that they had to rate valence and arousal of the presented pictures. After the acquisition, the investigator entered the chamber to start the tDCS. The extinction phase began automatically 10 min after stimulation onset. In the end, the participants received their allowance expense and were discharged.

### Statistical analysis

For statistical analysis of SCR data, GEEs were performed with SPSS (IBM Corp. Released 2017. IBM SPSS Statistics for Windows, Version 24.0. Armonk, NY: IBM Corp). GEEs are particularly recommended for analyses with correlated residuals and thus appropriate for longitudinal analyses with repeated measures (Liang and Zeger, 1986). We wanted to compare our two experimental groups with each other and with our control subjects in one statistical model, thus, we chose a two-factorial between-subject design and used two experimental and two sham groups. Stimulus (CS+ vs. CS–) and time (different factor steps for each model) were used as repeated within-subject factors and current flow direction (right vs. left anodal) and stimulation group (sham- vs. real-stimulation) as between-subject factors. To assess conditioning, the factor time consisted of two steps, one for the last two habituation trials and one for the last two acquisition trials. For analysis of early extinction processes, an initial model with the above-listed factors was done. The factor time included the last two acquisition trials as intercept and all regressors of the first extinction block (trial 1+2, 3+4, 5+6) as comparative values were created. To examine more precisely when the effect took place, further GEEs the same factors were built. These compared the regressor of the last two acquisition trials to every early extinction regressor in single models. We started with the first two extinction trials and moved on with the following trial-pairs in chronological order. After our adoption that tDCS will influence most notably early extinction learning, we stopped the model calculation as soon as one of the models did not show a significant stimulation effect anymore. Our hypotheses were limited to early extinction learning but because data for late extinction learning had been collected, we explored these late extinction trials, too. This explorative analysis was performed analogically to the analysis of early extinction processes, but with the last regressor of the first extinction block as intercept and all regressors from the second extinction block (trial 7+8, 9+10, 11+12) as comparative values.

Subjective ratings, state anxiety and affect were analyzed using GEEs again with stimulus (CS+ vs. CS–) and time (different factor steps for each model) as repeated within-subject factors and current flow direction (right vs. left anodal) and stimulation group (sham- vs. real-stimulation) as between-subject factors. To assess conditioning and CS-US-contingency awareness, the valence-, arousal-, and CS-UCS-contingency ratings after habituation and after the second acquisition block were compared. Analysis of early extinction processes was done by comparing the rating after the second acquisition block with the rating after the first extinction block. This was only possible for valence and arousal because CS-UCS-contingency. State anxiety and affect were only questioned after the acquisition and the second extinction block, so we could only analyze the reaction changes over the whole extinction course. Similar to the SCR analysis, we again did an explorative analysis of late extinction processes for valence and arousal ratings by comparing the ratings after the first and second extinction block in further GEEs as well.

Significant effects were defined with α ≤ 0.05, all tests were two-sided. The *post-hoc* analysis of significant GEEs outcomes was done with *t*-tests for independent or paired samples.

## Results

### Conditioning

Successful conditioning was reflected in a significant time x stimulus interaction for the last habituation regressor (trial 3+4) and acquisition regressor [trial 11+12; *wald*-χ2_(1, 336)_ = 43.60, *p* < 0.001]. For the between subject factors stimulation group and current flow direction no significant main effects or interactions could be found, suggesting that conditioning processes ran equal in all groups. *Post-hoc* paired-sample *t*-tests revealed a significant increase of reaction on CS+ [difference = 0.64, *t*_(83)_ = 5.11, *p* = < 0.001] while the reaction on CS– decreased [difference = −0.30, *t*_(83)_ = −3.27, *p* = 0.002]. The increase of the CS+/CS– discrimination during acquisition [difference = 0.94, *t*_(83)_ = 6.43, *p* < 0.001] led to significant reaction differences for CS+ and CS– in the end of the acquisition phase [difference = 0.91, *t*_(83)_ = 9.18, *p* < 0.001].

In subjective ratings all evaluation modalities showed successful conditioning and awareness of the CS-UCS-contingency with significant time x stimulus interactions [valence: *wald*-χ2_(1, 336)_ = 94.24, *p* < 0.001; arousal: *wald*-χ2_(1, 336)_ = 93.88, *p* < 0.001, contingency: *wald*-χ2_(1, 336)_ = 206.48, *p* < 0.001]. *Post-hoc t*-tests revealed a valence decrease and an arousal increase for the CS+ [valence: difference = −1.70, *t*_(83)_ = −8.44, *p* < 0.001; arousal: difference = 2.24, *t*_(83)_ = 8.96, *p* < 0.001] and opposite rating changes for the CS– [valence: difference = 1.06, *t*_(83)_ = 6.15, *p* < 0.001; arousal: difference = −0.86 *t*_(83)_ = −4.09, *p* < 0.001]. Therefore, for valence and arousal a crucial increase of discrimination learning took place during acquisition [valence: difference = 2.76, *t*_(83)_ = 9.67, *p* < 0.001; arousal: difference = 3.10, *t*_(83)_ = 9.61, *p* < 0.001] and led to a significantly different rating for CS+ and CS– in the end of the acquisition phase [valence: difference = 2.63, *t*_(83)_ = 9.91, *p* = 0.002; arousal: difference = 3.05, *t*_(83)_ = 11.72, *p* < 0.001]. Further, participants became aware of the CS–UCS-contingency during acquisition with an increase of CS+ rating from 39.05 to 78.69% [*t*_(83)_ = 12.67, *p* < 0.001] and a decrease of CS– rating from 38.21 to 18.21% [*t*_(83)_ = −6.62, *p* < 0.001]. There were no group differences for valence and contingency ratings, only for arousal ratings a significant main factor for the current flow direction appeared [*wald*-χ2_(1, 336)_ = 4.37, *p* = 0.037]. The following data inspection revealed that both right anodal stimulated groups rated CS+ and CS– in average 0.5 points more arousing than the other groups.

### Extinction

**Figure 3:**
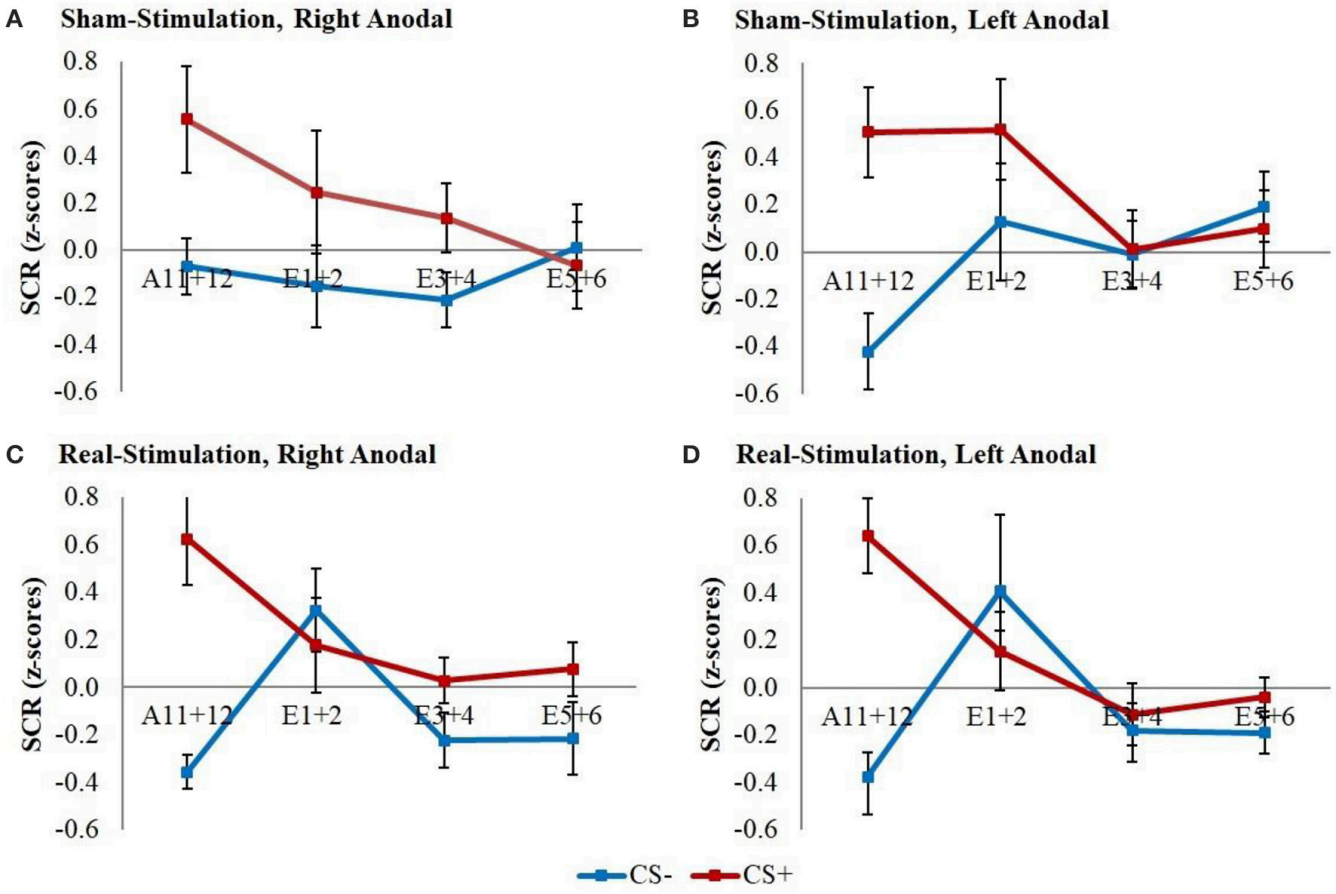
SCR during early extinction. Displayed are z-scored SCR values with their standard errors for early extinction learning separated for sham- and real-stimulated groups and both current flow directions. A11+12 = acquisition trials 11 and 12, E1+2 = extinction trials 1 and 2, E3+4 = extinction trials 3 and 4, E5+6 = extinction trials 5 and 6.

The GEE for the whole early extinction (see Figure 3) with the last acquisition regressor and all regressors of the first extinction block showed a significant time x stimulus x stimulation group interaction [*wald*-χ2_(3, 672)_ = 8.12, *p* = 0.044] while no effects on the current flow direction could be found. To narrow down the exact time point of the effect, the last acquisition regressor was then compared to each early extinction regressor in single GEEs starting with the regressor for trials 1+2 and moving on with the other regressors until the effect remained non-significant. Here, only the model with the first two extinction trials showed a significant time x stimulus x stimulation group interaction [*wald*-χ2_(1, 336)_ = 6.40, *p* = 0.011] with again no effects for the current flow direction.

**Figure 4:**
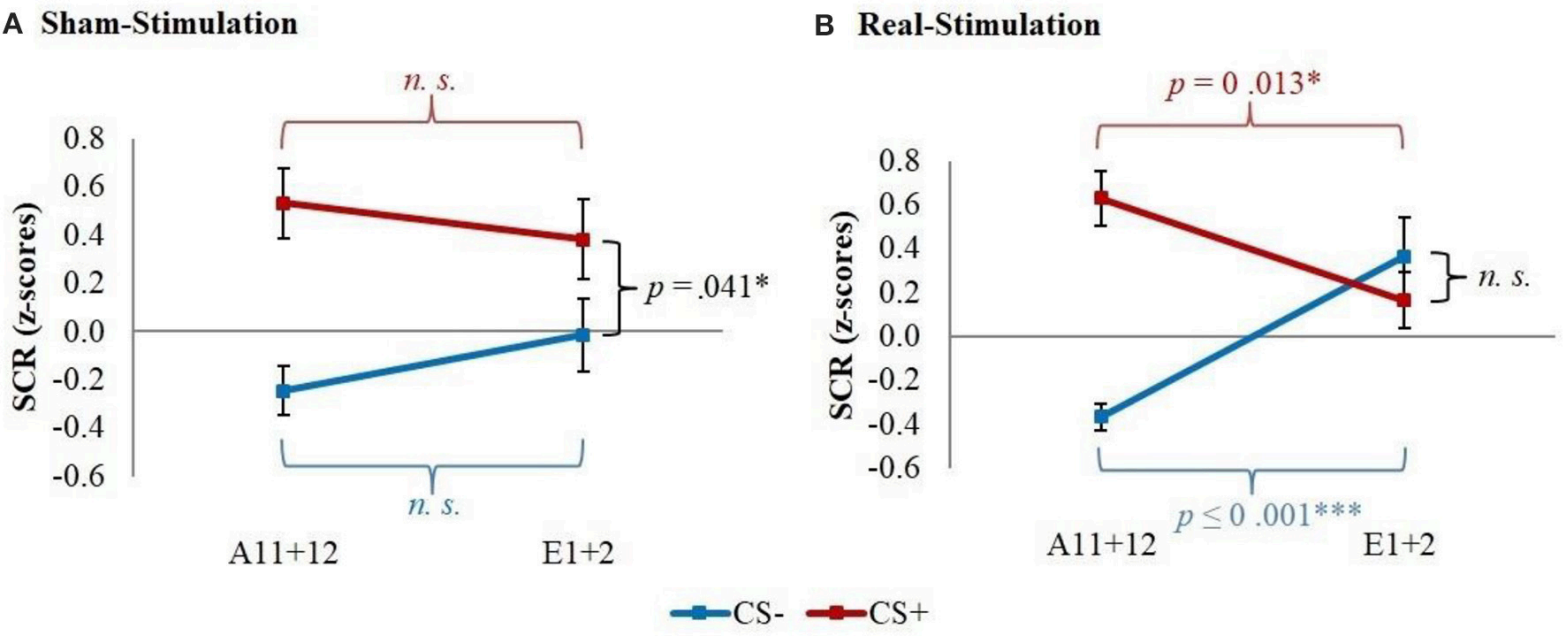
Improvement of early extinction learning. Displayed are z-scored SCR values with their standard errors for the last two acquisition (A11+12) and first two extinction trials (E1+2) for sham- and real-stimulated groups. Both real-stimulation groups showed a significant reaction decrease on CS+ and increase on CS– and a diminishing of CS+/CS– discrimination, whereas no significant reaction changes occurred in the sham-stimulation groups.

*Post-hoc t*-tests then revealed a significantly stronger decrease of CS+/CS– discrimination in both real-stimulation groups [difference = −0.82, *t*_(82)_ = −2.39, *p* = 0.019], but no significant group differences for reaction changes on CS+ and CS– separately. For a more precise background assessment of the discrimination loss, we performed paired-sample *t*-tests for the reaction changes on CS+ and CS– in real- and sham-stimulation groups individually. Here, the real-stimulation groups showed a significant decrease of CS+ reaction [difference = −0.47, *t*_(49)_ = −2.59, *p* = 0.013], but also an increase of CS– reaction [difference = 0.73, *t*_(49)_ = 3.99, *p* < 0.001]. Because of these reaction changes, the CS+/CS– discrimination diminished to a non-significant level after the first two extinction trials. In comparison there were no significant reaction changes in both sham-stimulation groups at all, so sham-stimulated participants still showed a relevant CS+/CS– discrimination after the first two extinction trials [difference = 0.40, *t*_(33)_ = 2.12, *p* = 0.041]. Thus, the CS+ reaction loss started earlier in both real-stimulation groups (see Figure 4).

**Figure 5:**
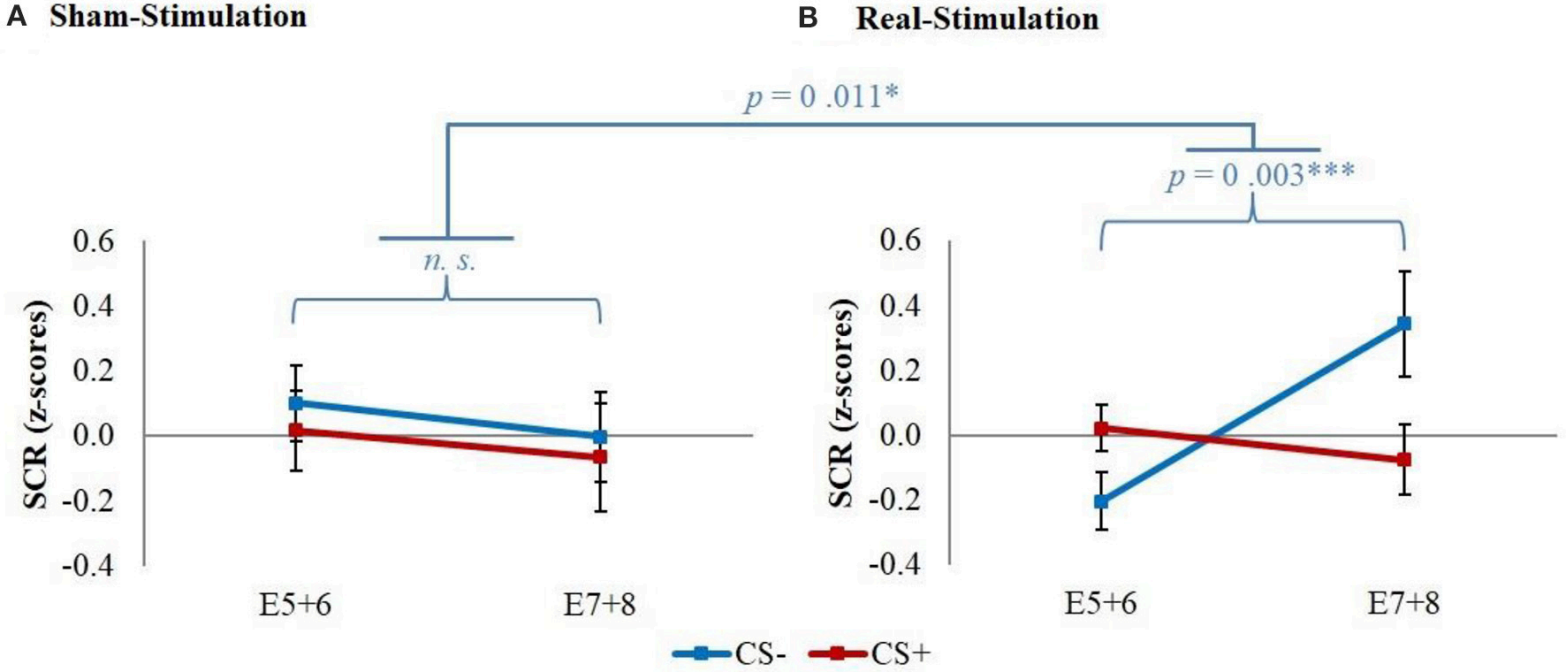
Initial CS– increase during late extinction in both real-stimulated groups. Displayed are z-scored SCR values with their standard errors for CS+ and CS– during the last two trials of the first extinction block (E5+6) and the first two trials of the second extinction block (E7+8). The real-stimulated groups showed a significantly higher increase of CS– reaction.

The explorative analysis of the late extinction processes showed a significant stimulation group x stimulus x time interaction for the whole second extinction block [*wald*-χ2_(3, 672)_ = 8.58, *p* = 0.035], which could be narrowed down temporally between the last regressor of the first and the first regressor of the second extinction block [*wald*-χ2_(1, 336)_ = 5.03, *p* = 0.025]. *Post-hoc t*-tests resulted in a short initial increase of CS– in both real-stimulation groups compared to the sham-stimulation groups [difference = 0.65, *t*_(82)_ = 2.59, *p* = 0.011] (see Figure 5).

**Figure 6:**
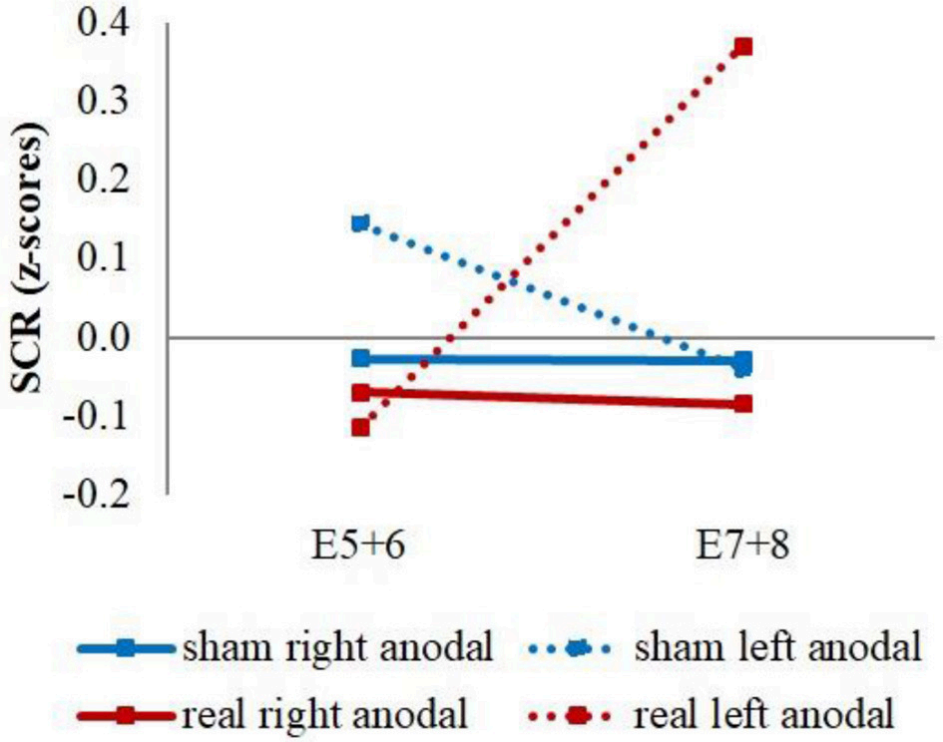
CS+/CS– average SCR values. Displayed are the averaged z-scored SCR-values over CS+ and CS– during the last two trials of the first extinction block (E5+6) and the first two trials of the second extinction block (E7+8) for sham- and real-stimulation groups and both current flow directions separated. The left anodal real-stimulated group showed a significantly higher CS+/CS– average increase than the right anodal real-stimulated and left anodal sham-stimulated groups.

Additionally, we had a significant stimulation group x current flow direction x time interaction between the last regressor of the first and the first regressor of the second extinction block [*wald*-χ2_(1, 336)_ = 4.16, *p* = 0.041]. This was caused by a stronger increase of the averaged reaction over CS+ and CS– for the left anodal real-stimulation group compared to the right anodal real-stimulation group [difference = 0.50, *t*_(48)_ = 2.15, *p* = 0.037] and left anodal sham-stimulation group [difference = 0.67, *t*_(39)_ = 2.72, *p* = 0.010] (see Figure 6).

For valence and arousal ratings no significant interactions for the stimulation group or current flow direction could be found, but both rating modalities showed significant stimulus x trials interactions [valence: *wald*-χ2_(1, 336)_ = 32.78, *p* ≤ 0.001; arousal: *wald*-χ2_(1, 336)_ = 10.71, *p* = 0.001], which revealed successful extinction processes in all groups alike. *Post-hoc t*-tests showed a significant increase in valence and decrease in arousal for the CS+ [valence: difference = 1.04, *t*_(83)_ = 6.86, *p* ≤ 0.001; arousal: difference = −0.89, *t*_(83)_ = −4.21, *p* < 0.001] and no significant rating changes for the CS–. Although the discrimination loss for both rating modalities was significant [valence: difference = −1.26, *t*_(83)_ = −5.91, *p* < 0.001; arousal: difference = 0.96, *t*_(83)_ = 3.44, *p* < 0.001], there were still substantial rating differences after the first extinction block [valence: difference = 1.37, *t*_(83)_ = 7.65, *p* ≤ 0.001; arousal: difference = −2.08, *t*_(83)_ = −10.07, *p* < 0.001]. Neither the explorative analysis of the second extinction block nor the analysis of the CS-UCS-contingency for the whole extinction yielded significant effects for the stimulation group or current flow direction.

**Figure 7:**
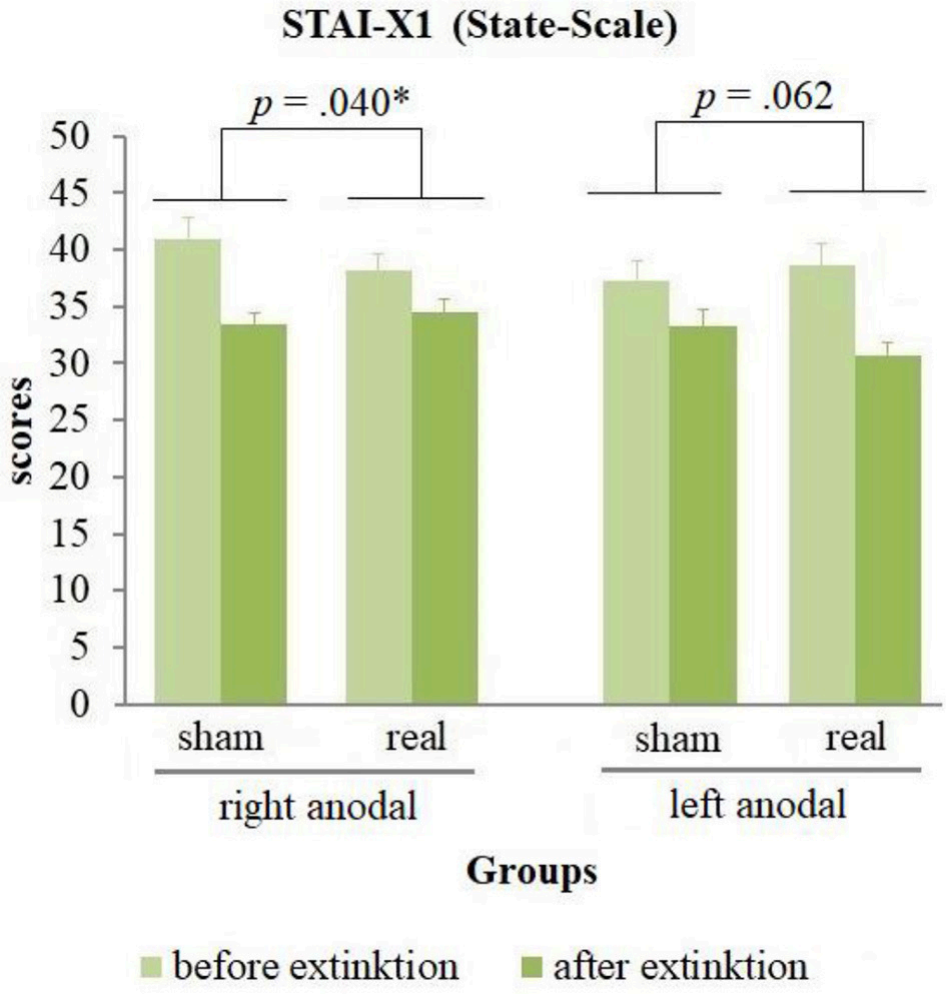
State anxiety before and after extinction. Displayed are the scores of the STAI-X1 questionnaires with their standard errors before and after extinction separated for both current flow directions and sham- and real-stimulation. The decrease of state anxiety was in right anodal stimulated participants significantly lower, in left anodal stimulated participants trend-significantly higher for real- compared to sham-stimulated subjects.

The analysis of the questionnaires (see Table 1) revealed no positive affect changes, but an equal decrease of negative affect in all groups during extinction [difference = −3.18, *t*_(82)_ = −7.88, *p* < 0.001]. Furthermore, a significant stimulation group x current flow direction x time interaction for the state anxiety [*wald*-χ2_(1, 167)_ = 8.58, *p* = 0.003] was found. A following breakdown of this three-way-interaction into a two-way-interaction for left and right anodal stimulated subjects separately revealed a significant stimulation group x time interaction for both current flow directions [right anodal: *wald*-χ2_(1, 86)_ = 4.41, *p* = 0.036; left anodal: *wald*-χ2_(1, 81)_ = 4.15, *p* = 0.042]. *Post-hoc* paired-sample *t*-tests showed a significant decrease of state anxiety in all 4 groups [right anodal real: difference = −3.58, *t*_(25)_ = −3.21, *p* = 0.004; right anodal sham: difference = −7.65, *t*_(16)_ = −4.64, *p* < 0.001; left anodal real: difference = −7.92, *t*_(23)_ = −5.18, *p* < 0.001; left anodal sham: difference = −4.38, *t*_(15)_ = −4.26, *p* = 0.001]. Further independent-sample *t*-tests compared the change of state anxiety from the rating before to the rating after extinction learning between real- and sham-stimulated subjects again for both current flow directions separately. These tests showed a significant lower decrease of state anxiety in real- compared to sham-stimulated subjects in the right anodal stimulated group [difference = −4.07, *t*_(41)_ = −2.12, *p* = 0.040]. In contrary, in the left anodal stimulated group real-stimulated subjects had compared to their sham-stimulated control group a trend-significant higher decrease of state anxiety during extinction [difference = 3.54, *t*_(38)_ = 1.92, *p* = 0.062; see Figure 7). Thus, right anodal tDCS attenuated the reduction of state anxiety during extinction learning. For an overview about the SCR data of all time points that were used for statistical analysis see Supplementary Figure 1.

## Discussion

The results of this study indicated a successful improvement of early extinction learning with a faster loss of CS+/CS– discrimination and an earlier decrease of reaction on CS+ in both real-stimulation groups. But a crucial limitation to this result is, that the faster CS+/CS– discrimination loss is not only driven by the reaction loss on CS+ but also by an unexpected initial increase of reaction on CS–. The additional explorative analysis of late extinction learning revealed a short initial increase of CS– reaction again at the beginning of the second extinction block in both stimulation groups. Contrary to our hypotheses, we found no differences in the current flow direction during early extinction learning. Only the explorative analysis of late extinction showed that the averaged reaction over CS+ and CS– had a higher increase between the end of the first and the beginning of the second extinction block in the left anodal compared to the right anodal real-stimulation group. Thus, according to this explorative analysis, right anodal current flow seemed to be the preferred direction at first glance. But on the contrary, questionnaires revealed a lower loss of state anxiety during extinction in exactly this right anodal real-stimulation group compared to its control group.

Surprisingly, extinction learning took place very fast and the participants needed only 4 extinction trials for a diminishing of their conditioned reaction. Thus, we had to correct our definition of “early extinction,” which was meant to take place during the whole first extinction block. As a temporal narrowing analysis was performed, we, however, recognized that our effect took place during the first half of actual extinction learning. Some other studies, that used similar conditioning paradigms with neutral looking faces as CSs and a scream as UCS, showed similarly short extinction learning phases. E. g. in Abend et al. (2016) CS+/CS– discrimination diminished during the first 4 and in Guhn et al. (2014) during the first 6 extinction trials (Guhn et al., 2014; Abend et al., 2016).

### Comparison to prior studies

The improvement of early extinction learning in this study resembles the effects that Guhn et al. (2014) could achieve by rTMS of the prefrontal cortex prior to extinction learning. Guhn's work did indeed lead our decision to expect tDCS effects notably during early learning processes. Another rTMS study by Raij et al. (2017) showed again a possible improvement of early extinction learning, but because of methodological manners, they could not state with certainty if their effect took place during early extinction learning or extinction recall.

So far, no study, that tried to modulate extinction via tDCS, had effects during early extinction learning. We cannot compare our effects to the extinction recall findings of Van't Wout et al. (2017) because we performed no extinction recall testing in our study. Between our effects and van't Wout's study in 2016, that indicated an improvement of late extinction learning, we do not see any parallels. But like Abend et al. (2016) we had a reaction increase on CS–. Compared to Abend's work, which showed a CS– increase during extinction recall, in our study short CS– increases at the beginning of every extinction learning block occurred. Because we did no extinction recall testing, we cannot make any statements about how the reaction on CS– could have developed during recall. As mentioned in the introduction, Abend et al. (2016) saw the unintentional stimulation of dorsomedial brain areas and their not temporally specific stimulation protocol as probable reasons for their results. We tried to prevent a CS– reaction increase by avoiding the stimulation of fear-generating dorsomedial brain areas, therefore, this does not seem to have caused the CS– increase. A temporally specific stimulation, in which the stimulation was started 100 ms after CS onset, could successfully improve extinction in rats (Milad et al., 2004) and in the rTMS approach of Raij et al. (2017). tDCS needs a fade-in and fade-out phase during which the current gets slightly ramped up and down, thus, it is not possible to perform such an exact timed stimulation protocol with tDCS. Guhn et al. (2014) did not use a temporally specific stimulation but could, however, improve extinction learning without a CS– increase. Therefore, other reasons might have caused this undesired side effect (see section Initial Reaction Increase on CS–).

With respect to these prior studies, there is some evidence that the modulation of extinction processes with brain stimulation may especially effect early extinction learning. Further, an impact of tDCS on CS– has occurred twice so far, thus, tDCS seems to enhance fear reactions on safety cues.

### Initial reaction increase on CS–

Besides the above-discussed causes for the unexpected short initial increase of reaction on CS–, another possible explanation is that our stimulation interfered with the safety information of the CS–, which is usually acquired during fear conditioning (Pavlov and Anrep, 1927; Rescorla, 1969). There is further evidence that supports this hypothesis. Firstly, our tDCS may have reached the amygdala, which is usually deactivated during safety learning (Schiller et al., 2008; Pollak et al., 2010). Secondly, tDCS did not affect the dorsolateral prefrontal cortex, which happens to be an important control area for safety learning processes (Pollak et al., 2010). Thus, maybe the stimulation led to a primary processing of extinction learning at the expense of safety information processing. This is compliant with the above-described input selective tDCS effect mechanisms. Thirdly, tDCS might have increased the dopaminergic secretion, which is beneficial for extinction learning (see Introduction), but affects safety learning unfavorably, as safety learning was increased under the use of dopamine D2 receptor antagonists (Pollak et al., 2008).

Another possible explanation for the CS- increase is fear generalization. This assumption was supported by a study of Kaczkurkin et al. (2017) who examined PTSD patients. PTSD patients usually generalize fear on an elevated level, thus, they show heightened fear responses to CS+ and to CS- in fear conditioning paradigms (Jovanovic et al., 2010; Norrholm et al., 2011). Kaczkurkin et al. (2017) found a flatter brain activity gradient between CS+ and CS- presentations in these patients in several brain areas. Some of these brain areas like the insula, the hippocampus, and the amygdala were located around the stimulated area in our study and could thereby be affected by the current flow as well. Our tDCS ran during CS+ and CS– presentations equally, thereby it could have led to a reduction of activity gradients between these two stimuli and to anxiety generalization processes.

A third explanation for this phenomenon is the fact that tDCS might have elevated the amount of sustained fear. Several fMRI studies demonstrated that sustained fear is associated with increased brain activity in the bed nucleus of the stria terminalis (BNST) and the insula (Munsterkotter et al., 2015; Herrmann et al., 2016b; Brinkmann et al., 2017). These brain areas were located around the area of our current flow as well, therefore, their affection could have elevated the level of sustained fear and created the reaction increase on CS–. The higher reaction increase on averaged CS+ and CS– in the left anodal real-stimulation group at the beginning of the second extinction block is compliant with this hypothesis, too. It has already been proven that right anodal tDCS of the lateral inferior frontal gyrus enhances emotional regulation processes and decreases anxiety reactions during sustained fear phases (Herrmann et al., 2016a). So, in our study, the right anodal stimulation could have led to a better control of sustained fear and thus countered the reaction increase.

### Effects of the current flow direction

Against our expectations, the current flow direction had no influence on the tDCS effects during early extinction learning. Only evidence from late extinction and state anxiety questionnaires revealed current flow direction effects.

Left anodal stimulated participants showed a higher initial increase on averaged CS+/CS– reaction in the second extinction block, so right anodal seemed to be advantageous at first glance. A possible explanation for this phenomenon could be that one brain area, which lies definitively nearer the activating effect of the anode during right anodal stimulation, namely the right lateral prefrontal cortex, is especially involved in emotional regulation processes as already said. Klumpers et al. (2010) revealed that the activity of the right lateral prefrontal cortex was correlated with downregulation of anxiety in a sustained fear paradigm. The activation of the right vmPFC, which is involved in the processing of negative emotions, was decreased simultaneously. Klumpers et al. (2010) concluded that the right lateral prefrontal cortex downregulates the right vmPFC and controls anxiety thereby. Herrmann et al. (2016a) could use the evidence from Klumpers's paper by increasing emotional regulation during a sustained fear phase through tDCS of the right inferior frontal gyrus. Thus, right anodal stimulation in our study could have increased emotional regulation, too.

But right anodal stimulation had some disadvantages as well. State anxiety decreased significantly less in the right anodal real-stimulated group and trend-significantly more in the left anodal real-stimulated group compared to their respective control groups, indicating that left anodal stimulation was the better choice. As there is some evidence that the right vmPFC is highly involved in the processing of negative emotions, one could imagine that a left-sided vmPFC activation could reduce anxiety better. In rat studies, a high rate of dopamine turnover, as well as a lesion of the right vmPFC, led to an increase in anxiety and stress. This indicates that dopamine reduces the activity of the right vmPFC and that the activity the right vmPFC increases the stress level (Thiel and Schwarting, 2001; Sullivan and Gratton, 2002). Consistent with this data from rats, human patients with right-sided vmPFC lesions showed in contrast to left lesioned patients, no anticipatory SCR in the Iowa gambling task, had an abnormal social behavior and problems with emotional processing (Tranel et al., 2002). Furthermore, a meta-analysis revealed a decreased left cortical activation in patients with an anxiety disorder (Thibodeau et al., 2006).

Therefore, the current flow direction can have different advantages or disadvantages. The decision which direction to prefer should be made with regard to the desired effects of the stimulation. Especially the use of right anodal tDCS should be taken with caution in anxiety patients in a clinical context as it seems to lower the loss of state anxiety compared to sham-stimulation during extinction learning.

### The mechanism for extinction improvement

We cannot say with certainty which of the above-presented tDCS effect mechanism was the main reason for the improvement of extinction learning in our study. The direct activation of the vmPFC, especially its left side, is a feasible option, but the elevation of the dopamine secretion, which is an important neurotransmitter for extinction processes, is a possible explanation, too. Furthermore, the activation of corticocortical pathways through tangential currents and thus an altered communication between several brain areas is worth considering as well.

In addition to the enhancement of extinction learning, tDCS could have elicited a more sensitive reaction to prediction errors and supported extinction learning thereby, too. The principle behind the concept of prediction errors is that learning arrives from the violation of expectations. Referring to extinction learning, this means that extinction is—at least partially—driven by the violation of the expectation that the UCS will occur after the CS+, which is a negative aversive prediction error. Thus, the main learning through prediction errors takes place at the beginning of the extinction phase, when the expectancy of the UCS is still strong (Rescorla and Wagner, 1972; Schultz and Dickinson, 2000; Niv and Schoenbaum, 2008). On the neuronal level, especially the transmitter dopamine is suspected to establish the link between extinction learning and prediction errors (Raczka et al., 2011; Berg et al., 2014).

There is some evidence which makes an involvement of enhanced prediction error processing in our study considerable. Firstly, our effect occurred only during very early extinction learning and thus during the period, in which the greatest prediction errors take place. Secondly, there was not only a very fast reaction loss on CS+ but also on CS– (after its initial increase). Classical extinction is generally understood as a relearning process of the CS-UCS-association (Bouton, 2002), thereby, it does not respond to reaction changes on the CS–, which was never paired with the UCS and thus acquired no association that could be relearned. However, through several possible mechanisms declared above, the CS– acquired a negative connotation in our study and—referring to prediction errors—this expectation of a negative outcome was then disappointed during the CS– presentations in the extinction phase. A feasible background mechanism of the enhanced prediction error processing in our study is that some brain areas, namely the vmPFC, middle temporal gyri and left lateral orbital gyrus, whose activity was also associated with prediction errors during extinction learning in a fMRI study by Spoormaker et al. (2011), were located around the current flow area of our tDCS as well. Thus, a tDCS induced activity increase in these areas could have mediated the effect on prediction error processing.

### Limitations

This study has some important limitations that require further research to state the effects of tDCS on extinction processes more clearly.

A first limitation is the sample size. Overall, we had 84 subjects for our analysis, which seems to be adequate at first glance, but considering the splitting into 4 groups made it rather modest. Further, about one-quarter of our participants had to be excluded due to insufficient conditioning. Exclusion rates in other studies, which used similar exclusion criteria, varied. E. g. Asthana et al. (2013) had to exclude only 14% of their subjects because of insufficient conditioning, whereas it was nearly 20% in Phelps et al. (2004) and Van't Wout et al. (2017). Lonsdorf et al. (2017) stated in her review that performance-based exclusion can easily lead to exclusion rates of over 50%. Additionally, despite blinding around 70% of our participants were able to evaluate their group assignment right. Poreisz et al. (2007), who investigated methodological manners regarding tDCS, declared a far lower detection rate with less than 20%. Our electrodes were applied on the face near the eye, where the skin is rather sensitive. This could be a reason for our high detection rate.

Secondly, the main stimulation target of this study, the vmPFC, plays a key role for extinction learning, consolidation, and recall (see Background), but it also seems to be important for the suppression of fear reactions. Several studies indicated that stimulation of the vmPFC led to a suppression of conditioned fear expression in rats (Milad and Quirk, 2002; Vidal-Gonzalez et al., 2006). The background mechanism of these reduced fear reactions can be explained by an improvement of extinction learning, but another explanation could be the simple reduction of fear expressions. Equally, we cannot definitively decide whether the tDCS in our study improved extinction learning or just suppressed fear expressions.

Another limitation is that tDCS started directly after the acquisition phase, so, it took place before the consolidation process of acquisition learning was completed. Some data suggest that extinction learning which takes place directly after conditioning has distinct neuronal mechanisms compared to extinction learning that is started after the completion of the consolidation of fear acquisition. For immediate extinction learning deleting processes seem to play a crucial role, whereas delayed extinction learning is rather a new associative learning process (Myers et al., 2006). But some of these data could not be replicated completely, thus, the data situation regarding this topic is still inconsistent (Herry et al., 2010; Lueken and Maslowski, 2012). Nevertheless, to prove the validity of our effects this study's tDCS protocol has to be replicated in a paradigm with fear conditioning and extinction learning on different days like it was e.g., in Abend et al. (2016) performed.

A further major limitation is that we did not implement an extinction recall testing. Thus, we cannot state if our effect is long-lasting, but the improvement of extinction learning can only have a positive effect on exposure therapies if the effect maintains between the therapy sessions. To make any clinical implications a testing of our tDCS protocol on extinction recall, like e.g., Abend et al. (2016) and Van't Wout et al. (2016, 2017), did, is necessary first.

Additionally, the effects of tDCS on extinction in our study were rather short and involved only the first two trials. But relatively speaking, the first two extinction trials represented the first half of the extinction learning process because after trial 4 the CS+/CS– discrimination vanished in our sham groups, too. But to ensure that our tDCS effects are strong and long-lasting enough to improve extinction learning in anxiety patients, which is typically a more complicated and protracted procedure than it is in healthy persons (Robinson et al., 2012), our stimulation protocol should be tested in these patients, too. Therefore, a direct testing during exposure settings would be useful because the long-term profit of this study was to find a tDCS protocol that can boost exposure therapy effects. Thus, the transfer of our effects to exposure therapy needs to be explicitly tested to assess whether a clinically relevant therapy improvement can be achieved.

But before this study's stimulation protocol should be tested in anxiety patients, further research is still needed. Besides our positive effects, we had a reaction increase on CS–, which is a crucial limitation for the results of this study. On the one hand it was jointly responsible for our main effect, the stronger CS+/CS– discrimination increase and on the other hand it could cause negative effects like a sustained fear increase, disruption of safety learning or anxiety generalization in patients. Therefore, the reason for the reaction increase on CS– needs to be explored. Additionally, some of our results indicate that the reduction of state anxiety during extinction learning is attenuated by right anodal tDCS. Subsequently, the tDCS protocol should be modified to prevent negative consequences for anxiety patients and ensure the safety of the stimulation.

## Conclusion

The current study has shown that tDCS with a bitemporal electrode positioning around the EEG positions F7 and F8 aimed to stimulate the vmPFC can improve extinction learning through a stronger CS+/CS– discrimination loss and a faster reaction decrease on CS+. But a crucial negative side effect, which also drove the CS+/CS– discrimination loss jointly, was an unexpected initial reaction increase on CS–. Such a reaction increase on CS– did not only occur in our study, it has already been observed by Abend et al. (2016). Thus, the background of this aspect should be investigated further. We assume that the interference with safety learning, fear generalization effects or the elevation of sustained fear are feasible reasons. Further, we discovered that the current flow direction had no effect during early extinction, but distinct advantages and disadvantages for the whole course of extinction. Left anodal stimulation led to a greater loss of subjectively rated state anxiety, whereas right anodal stimulation seemed to enhance emotional regulation. The intended stimulation effects and the anxiety extent of the stimulated population should thereby influence the decision which current flow direction to prefer.

Overall, the results of this study provide an important basis for the improvement of exposure therapies with tDCS. But for convincing remarks on the therapy success, the CS– increase and the transfer of our effects on anxiety patients in exposure situations must be explored first.

## Author contributions

ND and SH collected and analyzed data, ND drafted the paper. TP and MH designed the study, supervised the data collection and analyzes and revised the paper. All authors approved the final version of the paper.

### Conflict of interest statement

The authors declare that the research was conducted in the absence of any commercial or financial relationships that could be construed as a potential conflict of interest.

## Footnotes

^1^DGPPN, B., KBV, AWMF, AkdÄ, BPtK, BApK, DAGSHG, DEGAM, DGPM, DGPs, DGRW (Hrsg.) für die Leitliniengruppe Unipolare Depression (November 2015). “S3-Leitlinie/Nationale VersorgungsLeitlinie Unipolare Depression - Langfassung, 2. Auflage, Version 1.”).
