# Supplementary material for: Augmentation of Fear Extinction by Transcranial Direct Current Stimulation (tDCS)
Source: Front Behav Neurosci. 2018 Apr 25;12:76. doi: 10.3389/fnbeh.2018.00076 (PMC5996916; doi:10.3389/fnbeh.2018.00076)
Supplement: Supplementary file 1 [file Image_1.PDF]

Supplementary Material

Augmentation of fear extinction by transcranial direct current stimulation (tDCS)

N. Dittert, S. Hüttner, T. Polak[1], M.J. Herrmann*[1]

[1] Department of Psychiatry, Psychosomatics, and Psychotherapy, Center of Mental Health, University Hospital Würzburg, Margarete-Höppel-Platz 1, D – 97080 Würzburg, Germany

*** Correspondence:**
Prof. Dr. Martin Herrmann
Herrmann_m@ukw.de

1 Supplementary Data

None.

2 Supplementary Figures and Tables

2.1 Supplementary Figures

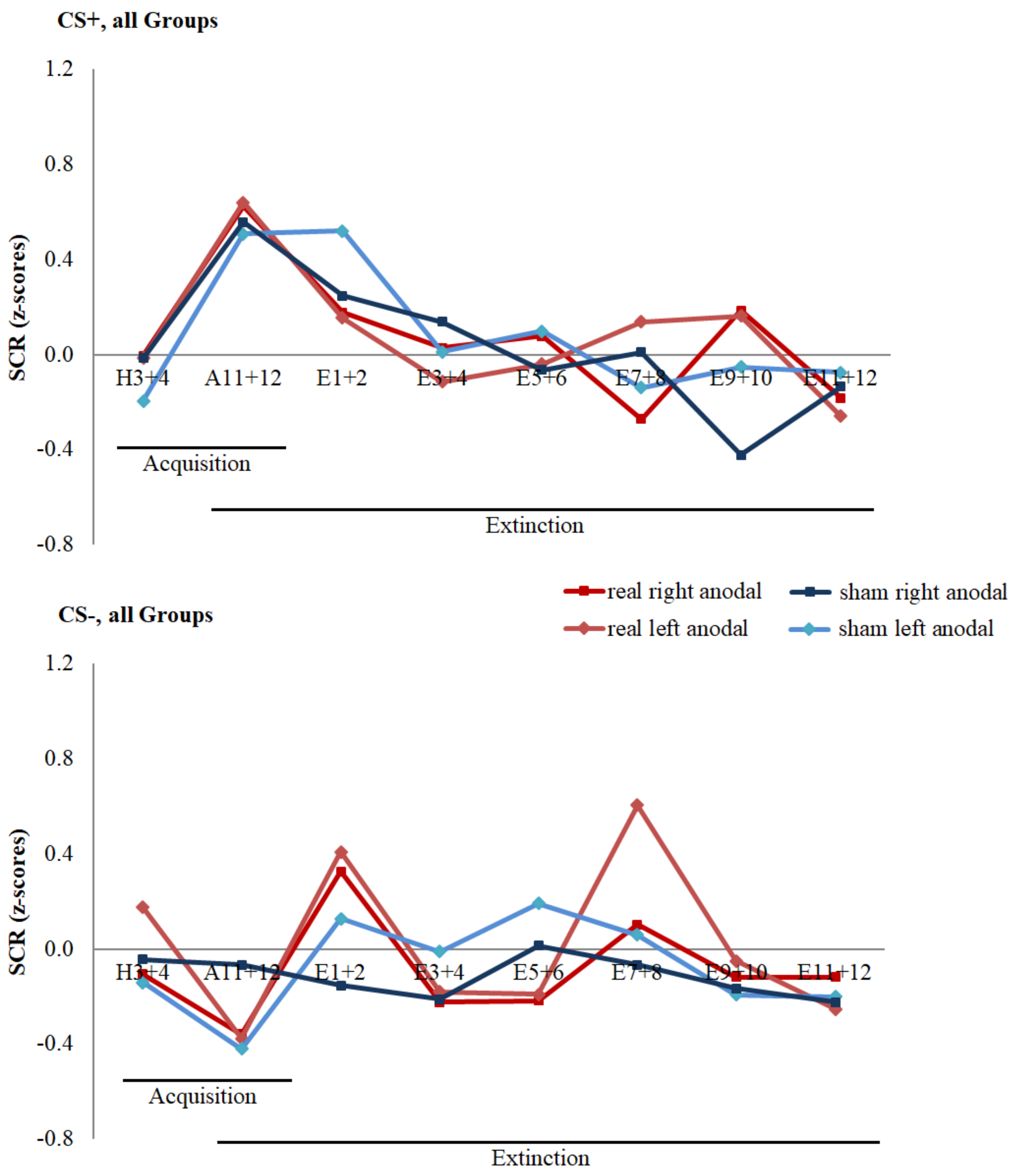

Supplementary Figure 1. The figure shows the z-scored SCR-values of all in the statistical analysis used regressors separately displayed for CS+ and CS-. H3+4 = habituation trials 3 and 4; A11+12 = acquisition trials 11 and 12; E1+2 = extinction trials 1 and 2; E3+4 = extinction trials 3 and 4; E5+6 = extinction trials 5 and 6; E7+8 = extinction trials 7 and 8; E9+10 = extinction trials 9 and 10; E11+12 = extinction trials 11 and 12.

2.2 Supplementary Tables

None.
